# Immunosuppression for immune-related adverse events during checkpoint inhibition: an intricate balance

**DOI:** 10.1038/s41698-023-00380-1

**Published:** 2023-05-12

**Authors:** Rik J. Verheijden, Mick J. M. van Eijs, Anne M. May, Femke van Wijk, Karijn P. M. Suijkerbuijk

**Affiliations:** 1 Department of Medical Oncology, University Medical Centre Utrecht, Utrecht University, Utrecht, The Netherlands; 2 Julius Center for Health Sciences and Primary Care, University Medical Centre Utrecht, Utrecht University, Utrecht, The Netherlands; 3 Center for Translational Immunology, University Medical Centre Utrecht, Utrecht University, Utrecht, The Netherlands

**Keywords:** Cancer immunotherapy, Cancer immunotherapy, Tumour immunology

## Abstract

Immune checkpoint inhibitors (ICIs) have changed perspectives for patients with cancer, but come with severe immune-related adverse events (irAEs). To prevent fatality or chronicity, these irAEs are often promptly treated with high-dose immunosuppressants. Until recently, evidence on the effects of irAE management on ICI efficacy has been sparse. As a result, algorithms for irAE management are mostly expert-opinion based and barely consider possible detrimental effects of immunosuppressants on ICI efficacy. However, recent growing evidence suggests that vigorous immunosuppressive management of irAEs comes with unfavourable effects on ICI efficacy and survival. With expansion of the indications of ICIs, evidence-based treatment of irAEs without hampering tumour control becomes more and more important. In this review, we discuss novel evidence from pre-clinical and clinical studies on the effects of different irAE management regimens including corticosteroids, TNF inhibition and tocilizumab on cancer control and survival. We provide recommendations for pre-clinical research, cohort studies and clinical trials that can help clinicians in tailored irAE management, minimising patients’ burden while maintaining ICI efficacy.

## Introduction

Immunotherapy is currently widely used in cancer patients. By blocking downregulators of the immune response, immune checkpoint inhibitors (ICI) can induce long-term tumour control and survival that may last for years after treatment discontinuation in some, but not all patients with cancer. Most commonly used immune checkpoint inhibitors target cytotoxic T lymphocyte antigen 4 (CTLA-4) or programmed cell death 1 (PD-1), whose mechanisms of action are extensively reviewed elsewhere^1,2^.

While augmenting immune responses, ICI also induce immune-related adverse events (irAEs). irAEs are graded according to the Common Terminology Criteria for Adverse Events (CTCAE) and range from mild (grade 1–2) to severe (grade 3–4) and are sporadically lethal (grade 5)^3,4^. Frequency of irAEs and most often affected organs differ according to ICI regimen and (to a lesser extend) cancer type (for example more vitiligo with melanoma). In patients treated with combined checkpoint inhibition (cICI) with CTLA-4 and PD-1 blockade (typically ipilimumab plus nivolumab), irAEs of any grade occur in more than 95% of patients with severe irAEs observed in ~60%^5^. With blockade of the PD-1/PD-L1 interaction, irAEs are less frequent, with ~65% of patients experiencing irAEs of any grade and 15% having severe irAEs^6^. Whilst resembling autoimmune diseases, irAEs generally have a more acute onset, are generally more severe and chronicity can mostly be averted if irAEs are promptly and adequately managed.

irAE management guidelines generally advise ICI interruption and sometimes corticosteroid initiation with grade 2 irAEs and definite ICI discontinuation with prompt initiation of immunosuppressants for grade 3 or higher irAEs to prevent fatality and chronicity^7–9^. Therapeutic corticosteroids (also referred to as glucocorticoids) such as prednisone and methylprednisolone are propagated as the first-line immunosuppressive treatment for almost all severe irAEs. When severe irAEs do not improve within 72 hours after corticosteroid administration, escalation of immunosuppression by increasing corticosteroid dosage and/or addition of other immunosuppressive therapeutics is indicated. The choice of second-line immunosuppressant is often based on experience in autoimmune diseases with close resemblance to the particular irAE, like inflammatory bowel disease (IBD) for ICI-induced colitis and rheumatoid arthritis (RA) for ICI-induced arthritis. In accordance with the vast experience in IBD, inhibition of tumour necrosis factor (TNF) is often considered as second-line irAE treatment, for example with the monoclonal antibody infliximab.

Due to the limited evidence on irAE management, guidelines are mainly expert opinion based, aiming at fast resolution of irAEs. Since evidence of possible detrimental effects of immunosuppressants on ICI efficacy has been limited until recently, these downsides of aggressive immunosuppression have generally not been considered in irAE management.

**Fig. 1:**
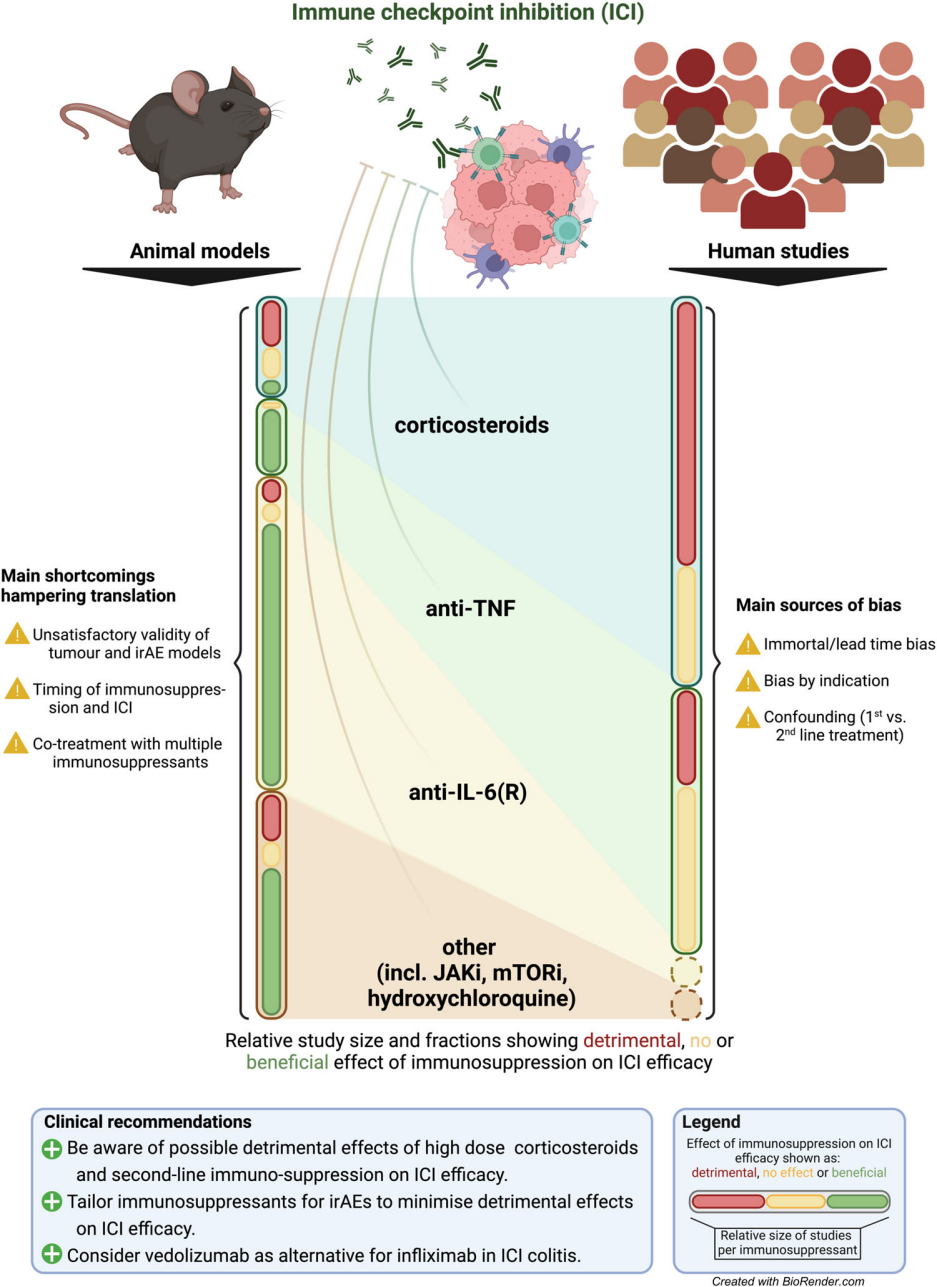
Main findings and limitations of animal models and human studies investigating impact of immunosuppressants on immune checkpoint inhibitor efficacy and consequential clinical recommendations. ICI immune checkpoint inhibition, irAE immune-related adverse event, TNF tumour necrosis factor, IL-6(R) interleukin-6(receptor), JAKi Janus kinase inhibitor, mTORi mammalian target of rapamycin inhibitor. Created with Biorender.com.

In this review, we discuss relevant pre-clinical and clinical data on immunosuppressants in the context of irAEs and ICI efficacy (Fig. 1). To ensure a complete overview of current literature, a thorough systematic literature search was conducted with synonyms for “immune checkpoint inhibition”, “immunosuppressive agents”, specific drug names and appropriate Mesh terms. By combining theoretical and empirical evidence, we provide guidance for clinical practice and future research.

## Immune-related adverse events and survival

Numerous reports have correlated irAE occurrence with increased overall survival^10–15^. These findings are prone to immortal-time bias (Box 1)^11^. Patients who develop an irAE after ICI initiation have guaranteed survival until irAE onset (therefore also called guarantee time bias or survivor bias), while patients who died without having an irAE might have developed one if they would not have died^16^. This may result in a biased estimation of survival differences in favour of the irAE group. However, several reports have shown that a correlation between irAEs and survival persisted when immortal-time bias was taken into account^11,13^. Interestingly, several studies have reported a survival benefit with low-grade, but not with high-grade irAEs^13,17–20^. This might be attributed to differences in ICI treatment duration, as ICI is often discontinued during high-grade, but not during low-grade irAEs. However, recent studies on neoadjuvant ICI demonstrate that responses occur early, with immune responses observed as early as 2 weeks after cICI initiation and pathological responses observed at 4–6 weeks^21–23^. Furthermore, ICI has been shown to induce long-term tumour control, even after discontinuation^24,25^. Since only 0.3–1.3% of ICI-treated patients have fatal irAEs^4^, it is highly unlikely that survival differences between low-grade and high-grade irAEs are due to fatal irAEs themselves. High-grade irAEs often require immunosuppression whilst low-grade irAEs do not. Therefore, survival differences between high-grade and low-grade irAEs could be an indication that immunosuppression impairs the initial survival benefit.

Box 1 Definitions of relevant biases Immortal-time bias arises in time-to-event analyses when the classifying event ( = exposure) occurs at some unspecified timepoint during follow-up. Subjects experiencing the classifying event (e.g. irAE) have a guaranteed immortal time until the occurrence of that event. Conversely, subjects who experience the outcome (e.g. death) early may not have had the time to experience the classifying event. Immortal-time is obvious when comparing survival of patients with irAEs to that of patients without. However, when comparing survival among patients with irAEs according to immunosuppressive treatment, immortal-time bias is minimised.

Confounding arises when an apparent effect of an exposure on an outcome is actually caused by another variable ( = confounder). A confounder has an association with both the exposure and the outcome and must not be the result of the exposure. For example, combined ICI is known to cause more (severe) irAEs which may be treated more vigorously than anti-PD-(L)1 monotherapy. Since type of ICI is also associated with survival, type of ICI rather than irAE management strategy might be (partially) accountable for a difference in survival if not correctly accounted for.

Confounding by indication is a specific form of confounding where the clinical indication for providing a certain treatment also affects the outcome. For example, if physicians tend to treat irAEs differently according to patient fitness, and patient fitness would also be correlated with survival, the association between irAE treatment and survival might be biased. While correcting for measured confounders reduces bias, residual confounding can remain in terms of unmeasured confounders.

Potential relevant confounders when assessing the impact of IS on survival are discussed in detail in the Supplementary Information.

## Corticosteroids

Corticosteroids have pleiotropic effects on various cells of the immune system^26–28^. Corticosteroids augment T_reg_ production and activity, inhibit T cell receptor (TCR) signalling, dampen T cell effector function, and induce a less pro-inflammatory cytokine profile^29–33^. Moreover, corticosteroids upregulate immune checkpoints such as CTLA-4, PD-1, TIM-3 and LAG-3 on T cells^29–31,34^.

Several mouse studies evaluating the effect of concurrent corticosteroid and ICI treatment on tumour growth and survival have demonstrated mixed results (Supplementary table 1)^30–32,35–38^. Four studies demonstrated a significantly increased tumour growth rate and/or impaired survival in mice treated with ICI plus dexamethasone compared to ICI therapy alone^30,32,35,38^, and one study showed non-significant trends in the same direction^36^. Conversely, Xiang et al. demonstrated better tumour control in mice treated with anti-PD-1 plus low-dose dexamethasone (0.1 mg/kg every 3 days) compared to anti-PD-1 alone in two tumour types^37^. Importantly, only two studies investigated corticosteroid administration after ICI initiation rather than concurrently, resembling the clinical situation of corticosteroid treatment for ICI-induced irAEs^35,38^. In a colon adenocarcinoma mouse model, Maxwell et al. observed that only 1 out of 8 anti-PD-1 plus dexamethasone treated mice had a complete response compared to 4/8 mice treated with anti-PD-1 alone with an increased tumour growth rate in the anti-PD-1 plus dexamethasone group^35^. Notably, two anti-PD-1 plus dexamethasone treated mice responded before dexamethasone initiation but progressed afterwards. Opposing results were observed by Tokunaga et al. who observed slightly but significantly impaired tumour control with high dose (2000 μg) but not low-dose (20 μg) concomitant dexamethasone, while no difference in tumour control was observed when dexamethasone was started after tumour regression^38^. Timing and dosing of corticosteroids may be crucial when assessing their impact on ICI efficacy and notably, inconsistencies exist between animal models and clinical practice. Overall, no strong conclusions can be drawn from animal studies with respect to the impact of corticosteroids for irAEs on ICI efficacy.

**Fig. 2:**
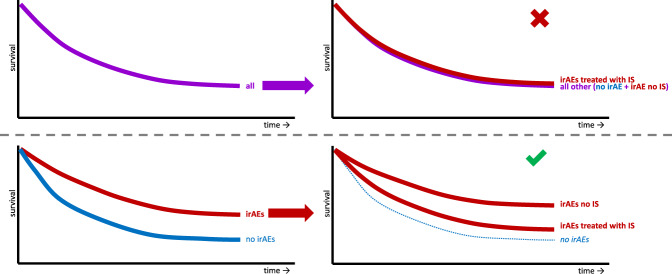
Fictitious Kaplan-Meier curves of ICI treated patients with or without immune-related adverse events (irAEs), treated with immunosuppressants (IS) or not. At baseline, it is unknown whether a patient will be treated with IS for irAEs. Comparing patients treated with IS for irAEs to all other patients (also those without irAEs) is likely incorrect. Under the assumption that patients with irAEs have a longer survival than patients without irAEs (lower left), survival of patients with irAEs treated with IS might appear similar to survival of all other patients (including those without irAEs; upper right), while it may actually be worse than survival of patients with irAEs which were not treated with IS (lower right). This is irrespective of whether the survival difference between patients with irAEs and those without is (partially) due to immortal-time bias.

Observational clinical studies on the association of corticosteroids with PFS and OS in ICI-treated cancer patients demonstrate contradictory results, originating from differences in indication and comparison group. Two meta-analyses concluded that patients receiving corticosteroids for cancer-related indications have worse survival, which might be a reflection of a worse prognosis due to bias by indication^39,40^. Conversely, both meta-analyses conclude that corticosteroids as irAE management do not seem to abrogate ICI efficacy. Most of the included studies compared patients whose irAEs were managed with corticosteroids to all other patients, including those without irAEs. This has two major limitations: First, results are prone to immortal-time bias, which may have resulted in an underestimation of a potential negative association between corticosteroid treatment and survival. Second, patients who develop irAEs are generally considered to have prolonged survival compared to patients who do not develop irAEs. Comparing survival of patients whose irAEs are managed with corticosteroids to all patients irrespective of irAE occurrence may obscure possible detrimental effects of corticosteroids for irAE management (Fig. 2). Indeed, all studies comparing corticosteroid-treated patients with irAEs to all other patients irrespective of irAE occurrence report no difference or a trend towards a favourable effect of corticosteroids on ICI efficacy (Supplementary table 2)^41–55^.

**Fig. 3:**
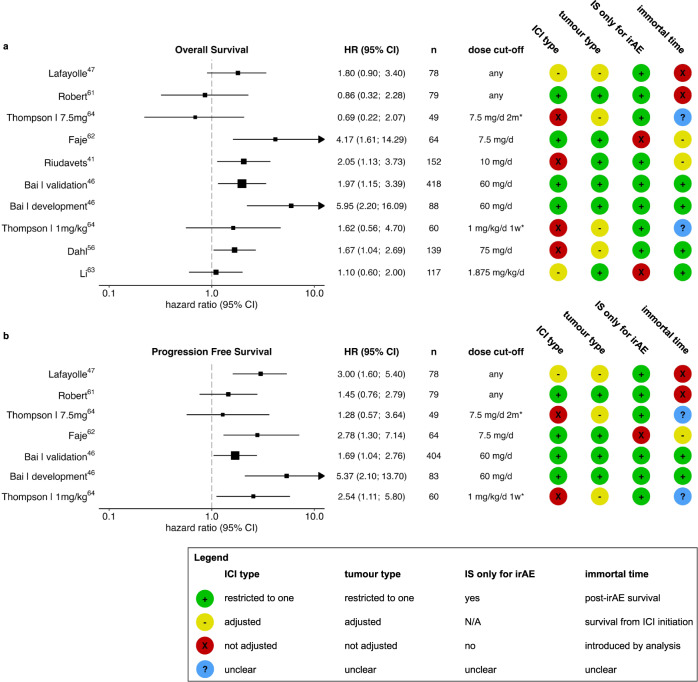
Survival in patients with immune-related adverse events (irAEs) with or without corticosteroid treatment. Overall survival (**a**) and progression-free survival (**b**) in patients with irAEs with or without corticosteroid treatment. Corticosteroid dose cut-offs represent prednisone equivalent dose. For immune checkpoint inhibitor (ICI) type and, tumour heterogeneity type, green (+) indicates restriction to one ICI regimen/tumour type, yellow (−) indicates adjustment for ICI regimen/tumour type and red (X) indicates that heterogeneity was not accounted for. In studies by Li et al. and Faje et al., corticosteroids irrespective of their indication (irAE or non-irAE) were included, which may have induced bias. In well-intended efforts to account for immortal-time bias, Robert et al. may have introduced bias rather than corrected for time to irAE onset and Lafayolle et al. have potentially overestimated the negative effect of corticosteroids on survival. Other studies accounted for immortal-time by analysing post-irAE survival (green +), did not account for difference in time-to-irAE onset (yellow −) or did not clearly describe start-point of survival analyses (blue?). *Thompson et al. compared two dosages of corticosteroids against the lowest dose of <7.5 mg/d for at least 2 weeks prednisone equivalent. HR hazard ratio for death, CI confidence interval, *n* number of participants in analysis, IS immunosuppressant, N/A not applicable.

Several studies making comparisons within the group of patients with irAEs, demonstrate a detrimental effect of high dose corticosteroids on ICI efficacy, while no unfavourable effect was observed in other studies (Fig. 3 and Supplementary table 3)^13,41,46,47,56–66^. Most of the latter studies reported on small groups or low-dose corticosteroid use during ICI irrespective of indication, or included multiple types of cancer or ICI which have different survival and toxicity profiles and are often unequally distributed among groups. In another analysis of randomised controlled trial data, Robert et al. reported no significant differences in PFS or OS among anti-PD-1-treated melanoma patients with irAEs treated with systemic corticosteroids compared to patients with irAEs without corticosteroid need^61^. The authors acknowledge the importance of immortal-time bias and the considerations regarding a landmark analysis when comparing survival of patients with irAEs to those without. However, applying this same landmark analysis in a context of two highly associated time-dependent variables (irAE onset and corticosteroid initiation) may have led to high dependency of survival outcomes on time of irAE onset relative to the landmark, possibly leading to obscured results.

In a retrospective study of anti-PD-1-treated melanoma patients with irAEs in two independent cohorts, Bai et al. examined the correlation between both PFS and OS with early high-dose corticosteroid exposure defined as 1-day peak dosage of ≥60 mg prednisone equivalent^46^. They assessed post-irAE PFS and OS in a landmark analysis in which only corticosteroid use within 8 weeks after anti-PD-1 initiation was considered and patients who had progressive disease or died within 8 weeks were excluded. Early high-dose corticosteroid exposure was strongly associated with worse post-irAE PFS and post-irAE OS in both cohorts. Similarly, in a cohort of infliximab-treated colitis patients with various tumour types and ICI regimens, patients with high peak dose of corticosteroids (≥75 mg prednisolone equivalent on 1 day) had a significantly worse overall survival than patient with lower dosages corticosteroids when corrected for tumour type and age, but not for type of ICI^56^.

Together, these data suggest that patients who received high-dose corticosteroids for irAEs have impaired survival. It is important to note that those patients who received the highest dosages of corticosteroids are likely also the ones who were treated with second-line immunosuppressants. Therefore, uncertainty remains whether the survival disadvantage is attributable to high-dose corticosteroids specifically, or merely reflects aggressive immunosuppression as a whole.

## TNF inhibitors

TNF is a molecule with both pro- and anti-inflammatory properties which are context-dependent^67–70^. In an effort to deduce these versatile immunological effects of TNF to a model summarising the effects of TNF inhibition in the context of ICI, Chen et al. inferred that short course TNF inhibition to treat irAEs presumably does not diminish ICI anticancer efficacy^67^, but this hypothesis has been questioned^71,72^. The complexity of TNF in the context of cancer is reflected by the opposing strategies to target TNF as a cancer therapy since its discovery. Early clinical trials using recombinant TNF in patients with advanced cancer demonstrated limited efficacy, accompanied with severe side effects^73–76^. Similarly, in early-phase clinical trials of TNF inhibitors in patients with advanced cancer, responses were rarely observed^77–80^.

Two mouse studies have shown that concomitant administration of TNF inhibition with ICI as upfront anti-cancer treatment not only resulted in less toxicity, but also in increased tumour control (Supplementary table 4)^81,82^. Bertrand et al. reported that simultaneous administration of TNF inhibition and anti-PD-1 6 days after tumour inoculation in mice led to tumour regression more often than anti-PD-1 alone with increased survival^81^. Similarly, Perez-Ruiz et al. demonstrated increased tumour control in mice treated with cICI and TNF inhibition 9 days after tumour inoculation compared to cICI alone^82^. This beneficial effect could not be confirmed in immunodeficient mice infused with human peripheral blood mononuclear cells, inoculated with a colon adenocarcinoma cell line^82^. Although no good animal models of spontaneous ICI toxicity currently exist, dextran sulphate sodium (DSS)-induced colitis severity was lower in mice treated with concomitant TNF inhibition and cICI than in those treated with cICI alone^82^. These results have led to the TICIMEL phase IB clinical trial in which advanced melanoma patients received a combination of upfront TNF inhibition (either infliximab or certolizumab) with cICI (NCT03293784)^83^. In total, ten out of 14 patients had a complete or partial response as best overall response, of whom five had an ongoing response after 1 year. Whether these data of upfront TNF inhibition are translatable to the clinical practice of irAE management after ICI initiation is uncertain.

**Fig. 4:**
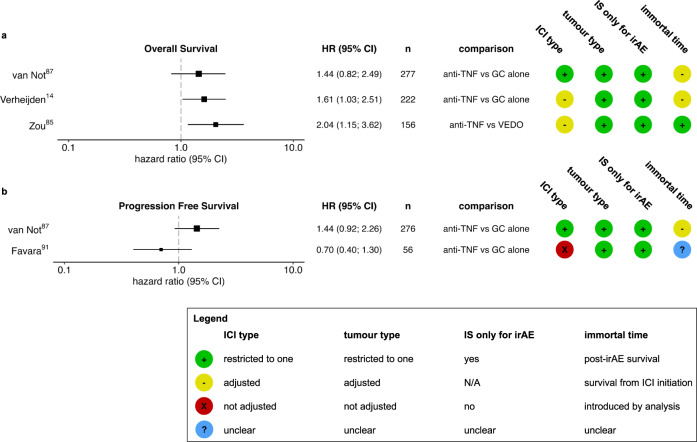
Survival in patients with immune-related adverse events (irAEs) with or without tumour necrosis factor (TNF) inhibition. Overall survival (**a**) and progression-free survival (**b**) in patients with irAEs with or without TNF inhibition. For immune checkpoint inhibitor (ICI) type and tumour, heterogeneity type, green (+) indicates restriction to one ICI regimen/tumour type, yellow (−) indicates adjustment for ICI regimen/tumour type and red (X) indicates that heterogeneity was not accounted for. Zou et al. analysed post-irAE survival which is theoretically less susceptible for difference in time-to-irAE (therefore green +), than survival from ICI initiation (yellow −, as in Fig. 2). Favara et al. did not clearly describe start-point of survival analyses (blue?). HR hazard ratio for death, CI confidence interval, *n* number of participants in analysis, IS immunosuppressant, GC glucocorticoid, VEDO vedolizumab, N/A not applicable.

Several cohort studies have reported on the association between the TNF inhibitor infliximab as second-line immunosuppressant for steroid-refractory irAEs and survival (Fig. 4 and Supplementary table 5)^14,59,65,84–94^. An early report on patients with anti-CTLA-4 induced colitis showed a non-significant trend towards improved survival in 7 patients who received infliximab compared to 22 who did not^88^. A similar trend was observed in PFS by Favara et al. in a group of 56 advanced melanoma patients with ICI induced colitis^91^. However, relatively more patients in the anti-TNF-treated group were treated with cICI. Other, larger studies did not find an improved survival with TNF inhibition or demonstrated diminished survival with TNF inhibition^14,65,87,92^. In a study of 222 first-line ICI-treated melanoma patients with grade 3 or higher irAEs, we found a shorter overall survival and cancer-related survival in patients treated with TNF inhibition as second-line immunosuppressant compared to patients who only required corticosteroid treatment. These results remained unchanged when adjusting for ICI regimen amongst other factors. Since ICIs were reintroduced as often in both groups, the survival difference was not explained by hesitance to reintroduce ICIs after steroid-refractory irAEs^14^. In a second study, in a more homogeneous cohort of 350 first-line cICI-treated melanoma patients with grade 3 or higher irAEs, we demonstrated that patients receiving second-line immunosuppression had worse OS, PFS and cancer specific survival compared with patients who only received corticosteroids^87^. Similar trends were observed for patients receiving second-line TNF inhibition and other second-line immunosuppressants^87^.

Most studies on escalated immunosuppression to treat steroid-refractory irAEs include patients with ICI-colitis or diarrhoea only. In these patients, vedolizumab proved an alternative with approximately similar effectiveness in terms of irAE resolution^85,95^. By blocking the interaction of α4β7-integrin with MadCAM-1, vedolizumab is considered to prevent T-cell homing in gastrointestinal tissue specifically, with the theoretical advantage of preventing local inflammation without diminishing systemic antitumor immunity. In a retrospective comparison of cancer patients who received infliximab, vedolizumab, or both as second-line immunosuppressants for ICI-induced diarrhoea or colitis, Zou and colleagues reported inferior response and post-irAE survival of infliximab-treated patients compared to vedolizumab-treated patients^85^. As the authors acknowledge, ICI efficacy was not the primary endpoint of this study, and the results could have been influenced by selection bias and confounding. Infliximab-treated patients received corticosteroids for a longer time period and more often had progressive disease before onset of toxicity (41% vs 24%). Interestingly, in the subgroup of patients without progressive disease at onset of colitis, proportionally more of the infliximab-treated patients had progressed at last follow-up compared to vedolizumab-treated patients (28% vs 13%) with similar follow-up duration.

Altogether, mouse models and clinical data on the effects of TNF inhibitors on tumours in the context of ICI are conflicting. Timing of TNF inhibition in relation to start of ICI and relative to corticosteroid initiation may be of major importance, reflecting the versatile role of TNF in different stages of cancer and immune activation. No definite conclusion can yet be drawn with respect to the initiation of TNF inhibitors in the context of irAEs. However, in patients with ICI-induced colitis, vedolizumab may be preferred over TNF inhibition when considering ICI efficacy.

## Interleukin-6 blockade

Interleukin (IL)-6 is a cytokine with a broad range of effects on immune and cancer cells^96–99^. High baseline IL-6 levels in serum and tissue and increase of serum IL-6 levels early on-treatment have been correlated to poor response and survival in ICI-treated cancer patients in several studies^100–104^. IL-6 can activate the Janus kinase (JAK) signal transducer and activator of transcription 3 (STAT3). IL-6 may induce angiogenesis and vessel permeability and lead to Th17 skewing. Activation of STAT3 has tumour promoting effects via suppression of apoptosis and tumour suppressors and induction of proliferation. Besides, STAT3 has immunosuppressive effects in the tumour microenvironment where its activation leads to inhibition of neutrophil, natural killer cell and effector T cell function, reduced DC maturation and expansion of T_reg_ and myeloid derived suppressor cells.

Several mouse studies have shown significantly (17 experiments) or non-significantly (4 experiments) improved tumour control or survival with upfront anti-IL-6 plus ICI compared to ICI alone (Supplementary table 6)^102,104–110^. This improved tumour control is presumably due to promotion of Th1-associated immunity^104,109^. Conversely, no improved tumour control or survival was observed in two mouse experiments^104,108^ and a slightly reduced anti-tumour effect was observed in the only two models of cICI^82^. Although upfront IL-6 blockade has been shown to mitigate experimental autoimmune encephalomyelitis symptoms^109^, no pre-clinical models have been reported that mimic the timing of immunosuppression in the context of irAEs in which IL-6 blockade is initiated after ICI.

Very limited clinical reports on the use of IL-6 blockade for irAEs do not show impaired ICI efficacy^111–114^. Stroud et al. reported on 87 nivolumab treated patients, of whom 34 patients received tocilizumab (anti-IL-6R) for severe irAEs^111^. When comparing tocilizumab treated patients with patients who did not receive tocilizumab, no difference in overall survival was observed. However, the exceptionally high number of tocilizumab treated patients (39%), and the inclusion of patients without irAEs in the comparison warrant caution when interpreting these results. Similarly, Dimitriou et al. report a median PFS of 6 months in 22 advanced melanoma patients receiving tocilizumab^113^, which is difficult to compare given the variety of ICI regimens patients received. The COLAR study (NCT03601611) demonstrated that 6 out of 20 patients treated with tocilizumab alone for ICI colitis or arthritis had progressive disease^114^. Although a 14 day period without any immunosuppression was required prior to tocilizumab initiation, 9 of the 20 patients had received systemic corticosteroids, including 3 who had also received infliximab for any irAEs prior to tocilizumab administration^114^. Preliminary results of an ongoing phase II trial of upfront tocilizumab plus cICI in advanced melanoma patients (NCT03999749) demonstrated a favourable overall response rate (ORR) within 12 weeks of 58% in 29 evaluable patients^115^. Another phase II trial of upfront tocilizumab plus cICI is currently underway (NCT04940299).

Taken together, in vivo data suggest that IL-6 blockade is a promising strategy in the context of ICI with suggested synergistic rather than detrimental effects on anti-tumour efficacy when given concomitantly. However, clinical data in the context of irAEs are too limited to draw conclusions on the effects of IL-6 blockade as irAE treatment on ICI efficacy.

## Other immune modulators

Other options for immunosuppression involve classical corticoid sparing disease modifying antirheumatic drugs (csDMARDs) such as methotrexate, mycophenolic acid, hydroxychloroquine and tacrolimus or kinase inhibitors such as JAK inhibitors and mammalian target of rapamycin (mTOR) inhibitors. Alternatively, intravenous immunoglobulins (IVIg), biologicals targeting other specific cytokines such as IL-1, IL-17 and IL-23 or targeting B-cells (such as rituximab) might be used to treat irAEs. Rationale for these therapeutics is reviewed elsewhere^116–118^.

In absence of clinical data, mouse studies entail the best available evidence for impact of JAK inhibitors^119–121^, mTOR inhibitors^122–125^, and hydroxychloroquine^126–128^ on ICI efficacy (Supplementary table 7). JAK1/2 loss-of-function mutations are associated with primary^129^ and secondary^130^ resistance to anti-PD-1. In contrast, multiple mouse models have demonstrated better tumour growth control with concomitant ICI and ruxolitinib (a JAK1/2 inhibitor) than with ICI alone^119–121^. Of note, one of these studies showed that only delayed administration of ruxolitinib (3 days after first anti-CTLA-4) provided better tumour control, which was not observed with simultaneous initiation^120^. A similar beneficial effect was found in mice treated with concomitant ICI with vistusertib (mTORC1/2 inhibitor) or sirolimus (mTORC1 inhibitor) compared ICI alone^122,123^. Interestingly, only addition of low-dose everolimus (mTORC1 inhibitor) to anti-PD-1 led to better bladder or cervical cancer control^124,125^, while addition of high-dose everolimus to anti-PD-1 showed a trend towards worse tumour control^124^. Three studies of hydroxychloroquine combined with anti-PD-1 in mice yielded contradictory results^126–128^. Sharma et al. observed better tumour control and survival in two melanoma models of simultaneous administration of anti-PD-1 and hydroxychloroquine^126^, while in a comparable model, Krueger et al. observed impaired tumour control when hydroxychloroquine was initiated 5 days after first anti-PD-1 initiation, again underlining the importance of timing^128^.

Clinical studies evaluating these agents upfront in combination with ICI are currently underway (NCT03681561, NCT03012230, NCT02423954, and NCT02890069). Although these agents have been studied in several pre-clinical models, these models lack translatability given difference in timing relative to ICI and dosage of immunosuppressants. Therefore, data are currently far too limited to draw any conclusions on the effects of these immune modulators on ICI efficacy in the context of irAEs.

## Discussion and future directions

Adequate immunosuppressive treatment of irAEs is crucial to prevent fatality and chronicity and to preserve quality of life. Since evidence on the association of immunosuppressants with ICI efficacy has been limited until recently, possible unfavourable effects on ICI efficacy are generally not addressed in current irAE management guidelines. This review indicates that aggressive immunosuppression to treat irAEs with high dose corticosteroids and escalated immunosuppression with other immunosuppressants may impair survival. Whether this effect is due to specific drugs or due to an overall effect of aggressive immunosuppression has yet to be determined. Nevertheless, we defined clinical recommendations based on pre-clinical and clinical data (Box 2).

Pre-clinical studies have currently mainly focussed on upfront combination of immunosuppressants with ICI, which hampers the translatability to the clinical setting of irAE management. Experimental models have clear limitations as models for human disease, including cancer and irAE, such as differences between immune systems^131^ and inoculation with allogenic cell lines instead of spontaneous tumour growth. Challenges with current irAE models have been critically reviewed elsewhere^132–135^. Some autoimmunity, like myocarditis, can reproducibly be induced in transgenic CTLA4^−/−^ or PDCD1^−/−^ mice^135^. In contrast to observations in humans, spontaneous irAEs, which are solely induced by immune checkpoint targeted monoclonal antibodies, are sparsely observed in rodents^133,136,137^. Therefore, inflammation is often induced by additional means in irAE experiments, either by using mice with a predisposing genetic background (such as non-obese diabetic mice), immunisation (as with experimental autoimmune encephalitis or myocarditis), irradiation or biochemical initiation of inflammation (as with DSS-induced colitis)^82,109,135,138^. In general, duration of most pre-clinical irAE research may be insufficient for genuine irAEs to develop^132^. This was illustrated by a study that induced overt colitis in Balb/c mice by prolonged (50 days) administration of anti-CTLA-4+/− anti-PD-1^139^. In addition, the relatively young age of mice used for experimental models is likely pivotal to why current models poorly reflect human irAEs. A recent study showed that anti-PD-1 treatment inflicted irAE-like infiltrates in various organs along with evidence for end-organ dysfunction in aged (>18 months) but not young (<3 months) tumour-bearing C57BL/6 and Balb/c mice^140^. Lastly, specific pathogen-free breeding conditions of laboratory mice limit translatability, since host microbiome composition has been associated with irAE development^141^. Some have attempted to address this limitation by skin commensal colonisation or faecal microbiota transfer followed by ICI administration to induce skin and gut irAEs^141,142^. A step further towards a diverse microbiome while maintaining the specific genetic background would be to exploit wildling mice, bred by transferring laboratory mouse embryos to wild female mice^143^.

Apart from the above inherent shortcomings in pre-clinical models, differences in timing of immunosuppressant administration relative to ICI initiation and dosage of immunosuppression may have led to inconsistent results across pre-clinical studies. Together, these differences may account for conflicting results observed in mice concurrently treated with ICI plus immunosuppression versus patients sequentially receiving immunosuppression for irAEs upon ICI. In Box 3 we present various considerations for pre-clinical irAE research and suggestions to improve current animal experiments. Such improvements to experimental models would indispensably come with rising costs, while essential deviations between mice and man will ultimately remain. Simultaneously, generation of human multi-omics data has become increasingly affordable and data availability through open repositories facilitates reuse by a wide public. These changes will further accelerate the era of ‘human immunology’. Herein, pre-clinical models will remain important, though no longer the go-to for investigating complex immunological disease such as irAEs and their interactions with environmental, tumour and host factors.

Previous clinical studies assessing the effects of immunosuppression for irAEs were mainly observational in heterogeneous cohorts with respect to cancer type and ICI regimen, often using a suboptimal control group. Therefore, most current clinical data are prone to confounding (by indication) and immortal-time bias. Randomised controlled trials (RCTs) comparing different irAE management regimens would provide clear answers to crucial questions. Although some clinical trials investigating irAE management are ongoing, none of them is sufficiently powered to assess ICI efficacy (Supplementary table 8). Given the vast heterogeneity in terms of cancer type, type and severity of irAEs and numerous possible immunosuppressants to test, RCTs powered on survival outcomes would demand tremendous multicentre efforts. Moreover, large well-structured collaborative observational studies can result in homogeneous and relevant cohorts, facilitating assessment of specific immunosuppressive regimens and validate results in different settings (Box 4).

Numerous questions remain to be answered in further well-designed pre-clinical and clinical studies. To guide clinical practice, data are needed to decipher whether impaired survival in patients treated with second-line immunosuppressants is due to specific therapeutics, reflects high dose corticosteroid usage or is a result of escalated immunosuppression as a whole. This could also provide clues for early versus late initiation of second-line immunosuppression and might be the steppingstone for skipping corticosteroids as first line and moving directly to more targeted immunosuppressants. Unravelling whether short course of high dose corticosteroids or lower dosages for a longer duration are most harmful is key to provide guidance for corticosteroid escalation and tapering strategies. Furthermore, whether associations between irAE treatment and ICI efficacy are similar for both early-onset and late-onset irAEs deserves further attention.

Taken together, clinicians should be aware of possible detrimental effects of immunosuppressants on ICI efficacy. Pre-clinical studies should consider timing and co-administration of several immunosuppressants at several dosages. Large multicentre randomised controlled trials and observational cohort studies are warranted to provide clinicians with more leads to effectively treat irAEs while maintaining ICI anti-tumour response.

Box 2 Clinical recommendations

- Adequate immunosuppressive treatment of irAEs is crucial to prevent fatality and chronicity and to preserve quality of life.
- Clinicians should be aware of possible detrimental effects of high dose corticosteroids and second-line immunosuppression on ICI efficacy.
- In patients experiencing ICI colitis, vedolizumab may be preferred over infliximab when considering ICI efficacy, but empirical evidence is weak.
- Tocilizumab during ICI is promising, but clinical data in the context of irAEs are currently lacking.
- Personalised immunosuppressive irAE management (as much as needed, as little as possible) minimises detrimental effects on ICI efficacy.

Box 3 Considerations for pre-clinical research

- Prolonged ICI treatment regimens (>3 weeks) could naturally induce some irAEs in mice.
- Using mouse-specific agents rather than human(sized) therapeutic antibodies could minimise neutralisation and enable prolonged antibody administration.
- The use of older mice (>20 weeks) can address age-related immune system changes.
- Introduction of immunosuppressants after ICI initiation rather than concomitantly in dosages used in irAE management would better reflect the clinical setting of irAE treatment.
- Corticosteroids as immunosuppressive backbone should be considered when designing pre-clinical studies on other immunosuppressants for irAE treatment.

Box 4 Considerations for clinical research

- Randomised controlled trials would be the ultimate method to answer outstanding questions.
- Collaborative, well-structured large observational studies can validate results in different settings of homogeneous populations and allow for multivariable analyses to address confounding (by indication).
- A careful consideration of immortal-time bias is warranted, especially with two closely related time-dependent events: irAE onset and immunosuppression initiation.
- Clinical studies should be restricted to patients with irAE (treatment), focus on post-irAE survival and consider adjustment for time to irAE onset.

## Methods

To ensure a complete overview of current literature, a thorough systematic literature search was conducted in PubMed with synonyms for “immune checkpoint inhibition”, “immunosuppressive agents”, specific drug names and appropriate Mesh terms, resulting in 7836 records. Relevant pre-clinical studies providing insight into pathophysiology of immunosuppression in the context of ICI and pre-clinical studies using animal models with ICI ± immunosuppression as determinant, and tumour volume or any measure of survival as outcome were selected. Clinical studies reporting on tumour response or survival in relation to immunosuppressive irAE management were selected, except for case reports and case series. Four relevant reports that were encountered through reference tracking were also included. To maximise comprehensiveness of this review, for immunosuppressants that yielded peer-reviewed (pre-clinical) studies through PubMed, we complemented our search with bioRxiv/medRxiv database screening for recently published manuscripts on the combined effect of immunosuppressants and ICI. Search strategies are displayed in Supplementary methods.

Main findings of mouse and human data are summarised in tables and if a hazard ratio with 95% confidence interval were reported, these were visualised in a forest plot per type of immunosuppressant. Adjusted hazard ratios from multivariable analyses were reported whenever possible. Most relevant and rigorous studies are discussed in detail in the main text. The majority of studies suffer from the same methodological pitfalls and generalisability issues and our outcome of interest was not the main endpoint of most clinical studies. Therefore, we have chosen to discuss these limitations and their implications for clinical practice in general and refrained from conducting a formal risk of bias assessment or meta-analysis.

### Reporting summary

Further information on research design is available in the Nature Research Reporting Summary linked to this article.

## Supplementary information

Supplementary Information

Reporting Summary

## Data Availability

Data referenced in this review can be accessed by following resources numbered in the Reference section.

## Code availability

The code to create Figs. 2 and 3 is available via: https://github.com/rjverheijden/ICI_immunosuppression_review_2023.
