# Supplementary material for: Immunosuppression for immune-related adverse events during checkpoint inhibition: an intricate balance
Source: NPJ Precis Oncol. 2023 May 12;7:41. doi: 10.1038/s41698-023-00380-1 (PMC10182067; doi:10.1038/s41698-023-00380-1)
Supplement: Supplementary file 1 — Supplementary Information [file 41698_2023_380_MOESM1_ESM.pdf]

Immunosuppression for immune-related adverse events during checkpoint inhibition: an intricate balance

Rik J Verheijden MSc[1,2*], Mick JM van Eijs MD[1,3], prof. dr. Anne M May PhD[2], prof. dr. Femke van Wijk PhD[3], prof. dr. Karijn PM Suijkerbuijk MD PhD[1]

Supplementary Information

- Supplementary methods
- Supplementary table 1: Mouse studies on correlation of corticosteroid administration with ICI efficacy
- Supplementary table 2: Clinical reports on the effects of corticosteroids for immune-related adverse events on ICI efficacy compared to all other patients irrespective of irAE occurrence
- Supplementary table 3: Clinical reports on the effects of corticosteroids for immune-related adverse events on immune checkpoint inhibitor efficacy
- Supplementary table 4: Mouse studies on correlation of TNF inhibition with ICI efficacy
- Supplementary table 5: Clinical reports on the effects of TNF inhibition for immune-related adverse events on immune checkpoint inhibitor efficacy
- Supplementary table 6: Mouse studies on correlation of interleukin-6 (receptor) inhibition with ICI efficacy
- Supplementary table 7: Mouse studies on correlation of Janus (JAK) kinase, mammalian target of rapamycin (mTOR) inhibition or hydroxychloroquine (HCQ) with ICI efficacy
- Supplementary table 8: Ongoing clinical trials on immunosuppression for irAE management

Supplementary Information

Supplementary methods

Potentially relevant confounders when assessing the impact of immunosuppression for immune checkpoint inhibitor (ICI) induced immune-related adverse events on survival may be:

- Type of ICI: Combined ICI is known to cause more (severe) irAEs which may be treated more vigorously than anti-PD-(L)1 monotherapy. Besides, type of ICI is associated with survival.
- Tumour type: Each cancer type and stage has its own prognosis. Tumour type may therefore strongly impact survival analyses. Although irAE profile may differ slightly according to tumour type (e.g. more often vitiligo with melanoma), its association with IS is unclear.
- irAE type: While irAE type is obviously correlated with its treatment (e.g. vedolizumab only for colitis), two studies observed no association between irAE type and survival[14,46].
- Other indications for IS: Other indications for corticosteroids include symptomatic brain metastases and as part of supportive care to increase patient comfort, which are both associated with a poor prognosis.
- Other immunosuppressants: patients treated with high dosages of corticosteroids are also more likely to receive second-line immunosuppressants and vice versa, which makes it difficult to differentiate between effects of specific immunosuppressants on ICI efficacy.
- ICI discontinuation and reintroduction: While guidelines propagate discontinuation of ICI when severe irAEs occur, they may be reintroduced more often with less severe irAEs. Therefore, patients with (more) IS might also have received less ICI.

Supplementary Information

PubMed search strategy

((("PD-1"[Title/Abstract] OR "PD1"[Title/Abstract] OR "programmed cell death 1"[Title/Abstract] OR "PD-L1"[Title/Abstract] OR "CTLA-4"[Title/Abstract] OR "CTLA4"[Title/Abstract] OR "cytotoxic T-lymphocyte-associated protein 4"[Title/Abstract] OR "CD152"[Title/Abstract]) AND ("anti*"[Title/Abstract] OR "inhibit*"[Title/Abstract] OR "blockade"[Title/Abstract])) OR "anti-PD-1"[Title/Abstract] OR "anti-PD1"[Title/Abstract] OR "anti-PD-L1"[Title/Abstract] OR "anti-PDL1"[Title/Abstract] OR "anti-CTLA-4"[Title/Abstract] OR "anti-CTLA4"[Title/Abstract] OR "pembrolizumab"[Title/Abstract] OR "nivolumab"[Title/Abstract] OR "cemiplimab"[Title/Abstract] OR "programmed cell death 1"[Title/Abstract] OR "durvalumab"[Title/Abstract] OR "avelumab"[Title/Abstract] OR "atezolizumab"[Title/Abstract] OR "ipilimumab"[Title/Abstract] OR "tremelimumab"[Title/Abstract] OR "ticilimumab"[Title/Abstract] OR "checkpoint inhibit*"[Title/Abstract] OR "immune checkpoint"[Title/Abstract] OR "Immune Checkpoint Proteins"[MeSH Terms]) AND ("Immunosuppressive Agents"[MeSH Terms] OR "Steroids"[MeSH Terms] OR "steroid*"[Title/Abstract] OR "corticosteroid*"[Title/Abstract] OR "glucocorticoid*"[Title/Abstract] OR "TNF"[Title/Abstract] OR "IL-6"[Title/Abstract] OR "cyclosporine"[Title/Abstract] OR "calcineurin"[Title/Abstract] OR "abatacept"[Title/Abstract] OR "adalimumab"[Title/Abstract] OR "anakinra"[Title/Abstract] OR "azathioprine"[Title/Abstract] OR "balsalazide"[Title/Abstract] OR "baricitinib"[Title/Abstract] OR "basiliximab"[Title/Abstract] OR "bimekizumab"[Title/Abstract] OR "brodalumab"[Title/Abstract] OR "budesonide"[Title/Abstract] OR "canakinumab"[Title/Abstract] OR "certolizumab"[Title/Abstract] OR "ciclosporine"[Title/Abstract] OR "cyclophosphamide"[Title/Abstract] OR "delgocitinib"[Title/Abstract] OR "dexamethasone"[Title/Abstract] OR "everolimus"[Title/Abstract] OR "etanercept"[Title/Abstract] OR "fedratinib"[Title/Abstract] OR "filgotinib"[Title/Abstract] OR "golimumab"[Title/Abstract] OR "guselkumab"[Title/Abstract] OR "hydroxychloroquine"[Title/Abstract] OR "infliximab"[Title/Abstract] OR "intravenous immunoglobulin*"[Title/Abstract] OR "ivig"[Title/Abstract] OR "ixekizumab"[Title/Abstract] OR "leflunomide"[Title/Abstract] OR "mesalazine"[Title/Abstract] OR "methotrexate"[Title/Abstract] OR "methylprednisolone"[Title/Abstract] OR "mycophenol*"[Title/Abstract] OR "natalizumab"[Title/Abstract] OR "obinutuzumab"[Title/Abstract] OR "oclacitinib"[Title/Abstract] OR "ocrelizumab"[Title/Abstract] OR "ofatumumab"[Title/Abstract] OR "olsalazine"[Title/Abstract] OR "perficitinib"[Title/Abstract] OR "pimecrolimus"[Title/Abstract] OR "predniso*"[Title/Abstract] OR "risankizumab"[Title/Abstract] OR "rituximab"[Title/Abstract] OR "ruxolitinib"[Title/Abstract] OR "sarilumab"[Title/Abstract] OR "secukinumab"[Title/Abstract] OR "siltuximab"[Title/Abstract] OR "sirolimus"[Title/Abstract] OR "sulfasalazine"[Title/Abstract] OR "tacrolimus"[Title/Abstract] OR "tildrakizumab"[Title/Abstract] OR "tofacitinib"[Title/Abstract] OR "tocilizumab"[Title/Abstract] OR "tralokinumab"[Title/Abstract] OR "triamcinolone acetonide"[Title/Abstract] OR "upadacitinib"[Title/Abstract] OR "ustekinumab"[Title/Abstract] OR "vedolizumab"[Title/Abstract] OR "vistusertib"[Title/Abstract]) AND "english"[Language] AND 2004/01/01:2022/11/17[Date - Publication]

Supplementary Information

Combined bioRxiv and medRxiv search strategies

1. Records posted between 1/1/2021 and 20/10/2022 were searched for:"checkpoint inhibitor HCQ" (8 hits) OR “checkpoint inhibitor hydroxychloroquine” (27 hits), all excluded based on title/abstract.
2. “checkpoint inhibitor mTOR” (534 hits), 2 screened on full text, all excluded.
3. “checkpoint inhibitor JAK” (431 hits), 9 screened on full text, 1 included in data synthesis.

Clinicaltrials.gov search strategies

(checkpoint AND (inhibitor OR blockade)) AND (corticosteroid OR prednisone OR prednisolone OR methylprednisolone OR dexamethasone) 152 hits

(pembrolizumab OR nivolumab OR cemiplimab OR durvalumab OR avelumab OR atezolizumab OR ipilimumab OR tremelimumab OR ticilimumab) AND (corticosteroid OR prednisone OR prednisolone OR methylprednisolone OR dexamethasone) 143 hits

(checkpoint AND (inhibitor OR blockade) OR pembrolizumab OR nivolumab OR cemiplimab OR durvalumab OR avelumab OR atezolizumab OR ipilimumab OR tremelimumab OR ticilimumab) AND (infliximab OR adalimumab OR budesonide OR vedolizumab OR tocilizumab) 70 hits

(pembrolizumab OR nivolumab OR cemiplimab OR durvalumab OR avelumab OR atezolizumab OR ipilimumab OR tremelimumab OR ticilimumab) AND (cyclosporine OR calcineurin OR abatacept OR anakinra OR azathioprine OR canakinumab OR certolizumab) 10 hits

(pembrolizumab OR nivolumab OR cemiplimab OR durvalumab OR avelumab OR atezolizumab OR ipilimumab OR tremelimumab OR ticilimumab) AND (ciclosporine OR everolimus OR etanercept OR hydroxychloroquine OR mesalazine) 31 hits

(pembrolizumab OR nivolumab OR cemiplimab OR durvalumab OR avelumab OR atezolizumab OR ipilimumab OR tremelimumab OR ticilimumab) AND (methotrexate OR mycophenol OR rituximab OR ruxolitinib OR secukinumab OR sirolimus) 95 hits

(pembrolizumab OR nivolumab OR cemiplimab OR durvalumab OR avelumab OR atezolizumab OR ipilimumab OR tremelimumab OR ticilimumab) AND (sulfasalazine OR tacrolimus OR tofacitinib OR ustekinumab) 2 hits

Supplementary table 1: Mouse studies on correlation of corticosteroid administration with ICI efficacy

Author	Type ICI	Mice	Immunosuppression	N_{mice}	Results	Effect
Maxwell 2018[35]	αPD-1	C57BL/6 mice<br>MC38 colon carcinoma<br>αPD-1 at d8, 10 and 12	DEX 10mg/kg/d concurrent (d8-12); late (d13-17); continuous with taper (d8->)	8	4/8 CR at d50 in anti-PD-1 alone vs 1/8, 1/8 and 0/8 in αPD-1 + DEX groups; ↑ tumour growth rate at d38 in αPD-1 + DEX (all groups) compared to αPD-1 alone	-
		C57BL/6 mice<br>GL261-luc glioma<br>αPD-1 d10, 12 and 14 after	DEX 10mg/kg/d concurrent (d8-12); late (d13-17); continuous with taper (d8->)	10	No significant differences in tumour control or survival between αPD-1 and αPD-1 + DEX mice	±
Giles 2018[31]	αCTLA-4	C57BL/6 mice<br>GL261-luc glioma<br>αCTLA-4 d13, 16 and 19	DEX 1mg/kg/d d7-50	7-8	↑ survival in αCTLA-4 + DEX vs αCTLA-4 alone although not formally tested and no ↑ survival in αCTLA-4 vs no treatment	(+)
Aston 2019[36]	αPD-1 + αCTLA-4	BALB/c mice<br>AB1 mesothelioma<br>cICI d12, 14, 16	DEX 1.25mg/kg/d d12-15	15	Non-significant trend towards ↓ tumour control and ↓ survival in cICI + DEX vs cICI alone	±/-
Tokunaga 2019[38]	αCTLA-4	BALB/c mice<br>CMS5a-NY-ESO-1<br>αCTLA-4 d3, 6, 9	DEX 20μg or 2000μg d3, 5, 7 **early**	5-11	↓tumour control in αCTLA-4 + high dose DEX vs αCTLA-4 alone; similar tumour control in αCTLA-4 + low dose DEX	-;±
	αCTLA-4	BALB/c mice<br>CMS5a-NY-ESO-1<br>αCTLA-4 d3, 6, 9	DEX 20μg or 2000μg d17, 19, 21 **late**	5-7	Similar tumour control in αCTLA-4 + low/high dose DEX vs αCTLA-4 alone	±
	αCTLA-4	BALB/c mice<br>CT26-NY-ESO-1<br>αCTLA-4 d3, 6, 9	DEX 20μg or 2000μg d3, 5, 7 **early**	4-10	Similar tumour control in αCTLA-4 + low/high dose DEX vs αCTLA-4 alone	±
	αCTLA-4	BALB/c mice<br>CT26-NY-ESO-1<br>αCTLA-4 d3, 6, 9	DEX 20μg or 2000μg d17, 19, 21 **late**	4-10	Similar tumour control in αCTLA-4 + low/high dose DEX vs αCTLA-4 alone	±

Author	Type ICI	Mice	Immunosuppression	N_{mice}	Results	Effect
	αPD-L1	BALB/c mice<br>CT26-NY-ESO-1<br>αPD-L1 d3, 6, 9	DEX 20µg or 2000µg d3, 5, 7 **early**	5-10	↓tumour control in αPD-L1 + high dose DEX vs αCTLA-4 alone; non-significant trend towards ↓tumour control in αPD-L1 + low dose DEX vs αCTLA-4 alone	-;±
	αPD-L1	BALB/c mice<br>CT26-NY-ESO-1<br>αPD-L1 d3, 6, 9	DEX 20µg or 2000µg d17, 19, 21 **late**	5-6	Similar tumour control in PD-L1 + low/high dose DEX vs αPD-L1 alone	±
Acharya 2020[30]	αPD-1 + αCTLA-4	C57BL/6 mice<br>MC38 colon carcinoma<br>cICI d7, 21, 35, 49 and 63	DEX 10mg/kg/d d7-17	9-10	↓tumour control in cICI + DEX vs cICI alone	-
Iorgulescu 2021[32]	αPD-1	C57BL/6 mice<br>GL261-luc2 glioma<br>αPD-1 d6, 9, 12, 15, (18, 21, 24 and 27)	DEX 1mg/kg/d, 2.5mg/kg/d and 10mg/kg/d d6-27	16-42	↓ tumour control and ↓ survival in αPD-1 + DEX vs αPD-1 alone, with increasing differences with increasing dose of DEX	-
Xiang 2021[37]	αPD-1	NOD/SCID mice<br>PBMC infusion d1 and 15<br>SW1990 pancreatic cancer injection d2<br>αPD-1 on d5, 12, 19 and 26	DEX 0.1mg/kg/3d d3-28	5	↑ tumour control in αPD-1 + DEX vs αPD-1 alone	+
		NOD/SCID mice<br>PBMC infusion d1 and 15<br>SGC-7901 gastric cancer injection d2<br>αPD-1 on d5, 12, 19 and 26	DEX 0.1mg/kg/3d d3-28	5	↑ tumour control in αPD-1 + DEX vs αPD-1 alone	+

N_{mice}: number of mice per group; αPD-1: programmed cell death 1 blockade; αCTLA-4: cytotoxic T lymphocyte-4 blockade; ICI: immune checkpoint inhibitor; cICI: combined ICI; DEX: dexamethasone

Supplementary table 2: Clinical reports on the effects of corticosteroids for immune-related adverse events on ICI efficacy compared to all other patients irrespective of irAE occurrence

Author	Type ICI	Cancer	Design	N_{CS}/N_{total}	Results	Effect
Riudavets 2020[41]	αPD-(L)1 ± αCTLA-4 /chemo	NSCLC	OC; average ≥10mg/d prednisone eq for irAEs vs all pts with <10mg/d	63/267	OS p=0.314	±
Bai 2021[46]	αPD-1	Melanoma	OC; irAEs with peak dose ≥60mg/d prednisone eq vs all other (as time-varying variable to account for immortal time bias)	29/90<br>90/419<br>29/90<br>90/419	OS $HR_{adj,dev}$ 1.29 (0.72-2.33)<br>OS $HR_{adj,val}$ 0.82 (0.57-1.18)<br>PFS $HR_{adj,dev}$ 1.79 (1.05-3.06)<br>PFS $HR_{adj,val}$ 1.29 (0.95-1.75)	±/-
Mouri 2021[42]	αPD-(L)1	NSCLC	OC; within primary non-progressors (PD <6m excluded); ≥10mg≥2w CS for irAEs vs all other	44/126	mOS 35.0 vs 41.0m (p=0.28)<br>mPFS 11.7 vs 16.0m (p=0.037)	±/-
Johnson 2015[48]	αCTLA-4	Melanoma	OC; CS for irAEs vs all other	12/34	OS no difference (p=0.31)	±
Horvat 2015[49]	αCTLA-4	Melanoma	OC; CS for irAEs vs all other	80/262	OS no difference (p=0.97)<br>TTF improved with CS (p=0.07)	±/+
Maher 2019[50]	αPD-(L)1	UCC	Pooled RCTs; within responders; CS for any indication vs no CS	84/351	No difference in duration of response HR 1.09 (0.70-1.69)	±
Vitale 2020[51]	αPD-1	RCC	OC; CS for irAE vs all other but only 85 of total 167 pts included	33/85	OS HR 0.58 (0.26-1.29)<br>PFS HR 0.66 (0.36-1.22)<br>ORR OR 1.45 (0.54-3.88)	±/+

Author	Type ICI	Cancer	Design	N_{CS}/N_{total}	Results	Effect
					DCR OR 1.22 (0.48-3.11)	
Shafqat 2018[52]	αPD-(L)1	Solid tumours	OC; CS for irAE vs all other	21/157	PFS HR 0.383 (0.160-0.918)	±
Arheden 2019[53]	αPD-1	Melanoma	OC; CS for irAE vs all other	25/130	OS HR 0.59 (0.30-1.19)	±/+
Skribek 2021[54]	αPD-(L)1 ± chemo	NSCLC	OC; CS for irAE vs all other	31/135	OS and PFS no difference (p=0.308 and p=0.380)	±
Gaucher 2021[55]	ICI	Solid tumours	OC; CS for irAE vs no CS for any indication	21/316	OS HR 1.01 (0.56-1.80)<br>ORR OR_{adj} 0.69 (0.52-6.56)	±
Ishihara 2021[43]	αPD-1 + αCTLA-4	RCC	OC; CS for irAE vs all other	7/59	1yOS 83.3% vs 87.0% (p=0.968)<br>mPFS 8.32 vs 12.2m (p=0.638)	±
Lafayolle de la Bruyère 2021[47]	ICI	Solid tumours	OC; CS for ≥gr3 irAE vs all other	45/864	OS HR_{adj} 0.99 (0.66-1.5)<br>PFS HR_{adj} 1.3 (0.91-2.0)	±
Paderi 2021[44]	αPD-(L)1	Solid tumours	OC; CS for irAE vs all other	41/146	PFS no difference (p=0.161)	±
Weber 2009[45]	αCTLA-4	Melanoma	RCT; **upfront** αCTLA-4+**budosenide** vs αCTLA-4 alone (similar irAE rates)	58/115	BORR 12.1% vs 15.8%<br>DCR 31% vs 35%<br>mOS 17.7 vs 19.3m	±

αPD-(L)1: programmed cell death (ligand) 1 blockade; αCTLA-4: cytotoxic T lymphocyte-4 blockade; ICI: immune checkpoint inhibitor; NSCLC: non-small cell lung cancer; UCC: urothelial cell carcinoma; RCC: renal cell carcinoma; OC: observational cohort study; RCT: randomised controlled trial; irAE: immune-related adverse event; CS: corticosteroid; PFS: progression free survival; OS: overall survival; mOS: median OS; TTF: time to treatment failure; (B)ORR: (best) objective response rate; DCR: disease control rate; HR: hazard ratio; OR: odds ratio; adj: adjusted

Supplementary table 3: Clinical reports on the effects of corticosteroids for immune-related adverse events on immune checkpoint inhibitor efficacy

Author	Type ICI	Cancer	Design	$N_{\uparrow CS}/N_{total}$	Results	Effect
Eggermont 2020[13]	αPD-1	Melanoma; adjuvant	RCT; pts with irAEs without or <30d CS vs placebo; >30d CS for irAEs vs placebo	63/190	RFS HR w CS 0.34 (0.21-0.56)<br>RFS HR wo CS 0.50 (0.23-1.07)	±/-
Bai 2021[46]	αPD-1	Melanoma	OC; within irAE; peak dose ≥60mg/d prednisone eq vs <60mg/d (post-irAE survival 8w landmark analysis)	12/88<br>29/418<br>11/83<br>26/404	OS $HR_{adj,dev}$ 5.95 (2.20-16.09)<br>OS $HR_{adj,val}$ 1.97 (1.15-3.39)<br>PFS $HR_{dev,val}$ 5.37 (2.10-13.70)<br>PFS $HR_{adj,val}$ 1.69 (1.04-2.76)	-
Dahl 2022[56]	ICI	Solid tumours	OC; within irAE treated with infliximab; start dose of CS tapering ≥75mg/d prednisolone eq vs <75mg/d; survival from first infliximab dose	65/139	OS HR_{adj} 1.67 (1.04-2.69)	-
Lafayolle de la Bruyère 2021[47]	ICI	Solid tumours	OC; within ≥gr3 irAE; any CS vs no CS; note: analyses may have led to overestimation of HRs	45/78	OS HR_{adj} 1.8 (0.9-3.4)<br>PFS HR_{adj} 3.0 (1.6-5.4)	±/-
Robert 2021[61]	αPD-1	Melanoma	Pooled RCTs; within non-progressors with irAEs <21w; with systemic CS for irAEs vs without CS; note: landmark analyses may have led to biased HRs	17/79	OS HR_{adj} 0.86 (0.32-2.28)<br>PFS HR_{adj} 1.45 (0.76-2.79)	±
Faje 2018[62]	αCTLA-4	Melanoma	OC; within hypophysitis; average ≤7.5mg/d prednsion eq in 2 months after hypophysitis diagnosis vs >7.5mg/d (irrespective of indication, but similar % with cranial radiotherapy)	50/64	OS HR_{adj} 0.24 (0.07-0.62)<br>TTF HR_{adj} 0.31 (0.12-0.70)<br>PFS HR 0.36 (0.14-0.77)	-

Author	Type ICI	Cancer	Design	$N_{\uparrow CS}/N_{total}$	Results	Effect
Li 2021[63]	αPD-1 ± αCTLA-4	Melanoma	OC; within ≥gr3 hepatitis; initial ≥1.5mg/kg/d methylprednisolone eq vs <1.5mg/kg/d (irrespective of indication); post-irAE survival	53/117	HR_{adj} OS 1.1 (0.6-2.0)	±
Riudavets 2020[41]	αPD-(L)1 ± αCTLA-4 /chemo	NSCLC	OC; within irAE pts; average ≥10mg/d prednisone eq vs <10mg/d	63/152	OS HR 2.05 (1.13–3.73)	-
Thompson 2021[64]	ICI	Solid tumours	OC; within colitis; average ≥1mg/kg/d ≥1w prednisone eq vs ≤7.5mg/d≥2m;<br>≥7.5mg/kg/d ≥2m vs≤7.5mg/d≥2m<br>(n=34 received αTNF±VEDO)	41/60<br><br>30/49	OS HR_{adj} 1.62 (0.56-4.70)<br>PFS HR_{adj} 2.54 (1.11-5.80)<br>OS HR_{adj} 0.69 (0.22-2.07)<br>PFS HR_{adj} 1.28 (0.57-3.64)	±/-
Wang 2018[65]	αPD-(L)1 ± αCTLA-4	Solid tumours	OC; within diarrhea; with CS±αTNF vs without CS±αTNF	79/117	OS no difference (all: p=0.232; only st IV: p=0.169)	±
Min 2015[66]	αCTLA-4	Melanoma	OC; within hypophysitis; >30mg/d >1w hydrocortisone eq vs ≤30mg/d for hypophysitis (n=5), other irAEs (8) or brain metastases (2)	15/25	12mOS 80% (SE 10.8%) vs 89% (SE 10.5%)	N/A
Paderi 2021[44]	αPD-(L)1	Solid tumours	OC; within steroid treated irAEs; peak >1mg/kg/d prednisone eq vs 0.5-1mg/kg/d and cumulative >500mg vs <500mg	16/41	PFS no difference (p=0.166 and p=0.578)	±
Romanski 2020[58]	ICI	Melanoma	OC; within hepatitis; >4000mg prednisolone vs <4000mg prednisolone	8/30	PD in 62.5% vs 22.7%	N/A
Gauci 2021[57]	ICI	Melanoma	OC; within hepatitis; with CS for irAE vs without	13/21	2yOS 56% vs 54% p=0.83	±
Dimitriou 2021[59]	ICI	Melanoma	OC; within irAEs; CS+αTNF/anti-IL6 vs CS only vs no CS within small subgroups according to therapy and setting	≤22/≤54	RFS/PFS and OS no difference (all p≥0.29)	±
Pan 2020[60]	αPD-1	Melanoma<br>NSCLC<br>RCC	OC; within irAEs; >10mg prednisone eq >2w vs >10mg prednisone eq <2w (irrespective of indication)	8/13<br>10/11<br>1/3	2yOS 37% vs 80%<br>2yOS 40% vs 0%<br>2yOS 100% vs 100%	N/A

$N_{\uparrow CS}/N_{total}$: number of patients in highest dosage group/total number of patients in analysis; αPD-(L)1: programmed cell death (ligand) 1 blockade; αCTLA-4: cytotoxic T lymphocyte-4 blockade; ICI: immune checkpoint inhibitor; chemo: chemotherapy; NSCLC: non-small cell lung cancer; RCT: randomised controlled

trial; OC: observational cohort study; irAE: immune-related adverse event; CS: corticosteroid; eq: equivalent; αTNF: tumour necrosis factor inhibition; VEDO: vedolizumab; RFS: recurrence free survival; PFS: progression free survival; OS: overall survival; mOS: median OS; PD: progressive disease; HR: hazard ratio; adj: adjusted; dev: development cohort; val: validation cohort

Supplementary table 4: Mouse studies on correlation of TNF inhibition with ICI efficacy

Author	Type ICI	Mice	Immunosuppression	N_{mice}	Results	Effect
Bertrand 2017[81]	αPD-1	C57BL/6 mice<br>B16K1 melanoma<br>αPD-1 d6, 10 and 13	αTNF 10mg/kg d6, 10, 13 and 16	24	↑ tumour control and ↑ survival in αPD-1 + αTNF vs αPD-1 alone	+
		Balb/c mice<br>4T1 breast cancer<br>αPD-1 d6, 9 and 13	αTNF 10mg/kg d6, 9 and 13	6	↑ tumour control in αPD-1 + αTNF vs αPD-1 alone	+
Perez-Ruiz 2019[82]	αPD-1 + αCTLA-4	C57BL/6 mice<br>MC38 colon carcinoma<br>cICI d9 and 15	αTNF 125μg or etanercept 40μg d9, 11, 13 and 15	12-23	↑ tumour control in cICI + αTNF/etanercept vs cICI alone no significant differences in survival	+/±
	αPD-1 + αCTLA-4	C57BL/6 mice<br>MC38 colon carcinoma<br>DSS induced colitis d9-11<br>cICI d9 and 15	αTNF 125μg or etanercept 40μg d9, 11, 13 and 15	11-17	↑ tumour control and ↑ survival in cICI + αTNF/etanercept vs cICI alone	+
	αPD-1 + αCTLA-4	C57BL/6 mice<br>B16-OVA melanoma<br>cICI d9 and 15	αTNF 125μg or etanercept 40μg d9, 11, 13 and 15	6	↑ tumour control and ↑ survival in cICI + αTNF/etanercept vs cICI alone	+
	αPD-1 + αCTLA-4	$Rag2^{-/-}Il2rg^{-/-}$ mice<br>HT29 colon carcinoma<br>PBMC infusion d7<br>cICI d7, 11, 14 and 17	etanercept 40μg d7, 11, 14 and 17	6	No significant difference in tumour control	±

N_{mice}: number of mice per group; αPD-1: programmed cell death 1 blockade; αCTLA-4: cytotoxic T lymphocyte-4 blockade; αTNF: tumour necrosis factor inhibition; cICI: combined immune checkpoint inhibition

Supplementary table 5: Clinical reports on the effects of TNF inhibition for immune-related adverse events on immune checkpoint inhibitor efficacy

Author	Type ICI	Cancer	Design	N_{IS}/N_{total}	Results	Effect
			**αTNF vs corticosteroids alone**			
Arriola 2015[88]	αCTLA-4	Solid tumours	OC; within diarrhoea; CS+αTNF vs CS alone	7/29	Trend towards ↑OS with (p=0.23)	±/+
Wang 2018[65]	αPD-(L)1 ± αCTLA-4	Solid tumours	OC; within diarrhoea; CS±αTNF vs CS alone (all stages; stage IV malignancies only)	35/79; 23/61	OS no difference or a trend towards ↑OS (p=0.768; 0.263)	±/-
Johnson 2018[89]	ICI	Melanoma	OC; within diarrhoea; CS±αTNF vs CS alone	18/40	OS no difference (p=0.741) TTF 9.0m (5.6-NR) vs 12.5m (5.8-NR)	±
Lesage 2019[90]	ICI	Melanoma	OC; OS and PFS of CS+αTNF for colitis vs historical control	27	mOS 12m mPFS 3m 1yOS 65.2%	N/A
Favara 2020[91]	ICI	Melanoma	OC; within gr3 diarrhoea/colitis; CS+αTNF vs CS alone	16/56	PFS HR 0.7 (0.4-1.3)	±/+
Nahar 2020[92]	ICI	Melanoma	OC; within colitis; CS+αTNF+other vs CS+αTNF vs CS alone	10 vs 38 vs 52	no differences in OS (p=0.73) trend towards ↓PFS in CS+αTNF±other vs CS (p=0.27);	±/-
Verheijden 2020[14]	ICI	Melanoma	OC; with gr3-4 irAE; CS+αTNF vs CS alone	65/222	OS HR_{adj} 1.61 (1.03-2.51)	-
Alexander 2021[93]	ICI	Solid tumours	OC; colitis requiring CS+αTNF	111	1yOS 83%	N/A
Araujo 2021[94]	ICI	Solid tumours	OC; irAE requiring αTNF	50	Median post-αTNF-initiation OS 13m (7.3-19.3)	N/A
Van Not 2022[87]	αPD-1 + αCTLA-4	Melanoma	OC; with gr3-4 irAE; CS+ αTNF vs CS alone	61/276	OS HR_{adj} 1.44 (0.84-2.49) PFS HR_{adj} 1.44 (0.92-2.26)	±/-
			**αTNF vs other sIS**			
Abu-Sbeih 2019[84]	ICI	Solid tumours	OC; within colitis; CS+αTNF±VEDO vs CS+VEDO	52/84	Trend towards ↓OS (p=0.151)	±/-

Supplementary Information

Author	Type ICI	Cancer	Design	N_{IS}/N_{total}	Results	Effect
Zou 2021[85]	ICI	Solid tumours	OC; within colitis; CS+αTNF vs CS+VEDO; post-irAE OS for all, genitourinary cancer, lung cancer and melanoma patients	94/156	$OR_{PD,adj}$ 5.24 (2.33-11.77)<br>OS_{all} HR_{adj} 2.04 (1.15-3.62)<br>↓$OS_{genitourinary}$ (p=0.032)<br>↓OS_{lung} (p=0.044)<br>↓$OS_{melanoma}$ (p=0.214)	-
Van Not 2022[87]	αPD-1 + αCTLA-4	Melanoma	OC; with gr3-4 irAE; CS+αTNF vs CS + other sIS	61/95	OS HR 0.99 (0.56-1.76)<br>PFS HR 1.17 (0.71-1.92)	±
			**sIS vs corticosteroids alone**			
Burdett 2019[86]	ICI	Solid tumours	OC; OS of CS+sIS vs historical control	19	mOS 9.4m	N/A
Dimitriou 2021[59]	ICI	Melanoma	OC; within irAEs; CS+αTNF/α IL6 vs CS only vs no CS within small subgroups according to therapy and setting	≤22/≤54	RFS, PF and OS no difference (all p>0.29)	±
Van Not 2022[87]	αPD-1 + αCTLA-4	Melanoma	OC; with gr3-4 irAE; CS+ sIS vs CS alone	115/350	OS HR_{adj} 1.54 (1.03-2.30)<br>PFS HR_{adj} 1.40 (1.00-1.97)	-

αPD-(L)1: programmed cell death (ligand) 1 blockade; αCTLA-4: cytotoxic T lymphocyte-4 blockade; ICI: immune checkpoint inhibitor; OC: observational cohort study; irAE: immune-related adverse event; CS: corticosteroid; sIS: second line immunosuppression; αTNF: tumour necrosis factor inhibition; VEDO: vedolizumab; αIL-6: interleukin-6 blockade; RFS: recurrence free survival; PFS: progression free survival; OS: overall survival; mOS: median OS; TTF: time to treatment failure; HR: hazard ratio; adj: adjusted; NR: not reached; N/A: not applicable.

Supplementary table 6: Mouse studies on correlation of interleukin-6 (receptor) inhibition with ICI efficacy

Author	Type ICI	Mice	Immunosuppression	N_{mice}	Results	Effect
Perez-Ruiz 2019[82]	αPD-1 + αCTLA-4	C57BL/6 mice<br>MC38 colon carcinoma<br>cICI d9 and 15	αIL-6 250μg d9, 11, 13 and 15	13-14	↓ tumour control in cICI + αIL-6 vs cICI alone	-
	αPD-1 + αCTLA-4	C57BL/6 mice<br>B16-OVA melanoma<br>cICI d9 and 15	αIL-6 250μg d9, 11, 13 and 15	12	↓ tumour control in cICI + αIL-6 vs cICI alone	-
Hailemichael 2022[109]	αCTLA-4	C57BL/6 mice<br>B16.BL6 melanoma<br>αCTLA-4 d3, 6 and 9	αIL-6 200μg d3, 5, 7 and 9	10	↑ tumour control and non-significantly ↑ survival in αCTLA-4 + αIL-6 vs αCTLA-4 alone	+/±
	αCTLA-4	C57BL/6 mice<br>B16.BL6 melanoma<br>EAE induction on d3<br>αCTLA-4 d3, 6 and 9	αIL-6 200μg d3, 6 and 9	10	Non-significantly ↑ tumour control in αCTLA-4 + αIL-6 vs αCTLA-4 alone	±
	αCTLA-4	Balb/c mice<br>CT26 colon carcinoma<br>αCTLA-4 d6, 8, 10 and 12	αIL-6 200μg d6, 8, 10 and 12	10	↑ tumour control and non-significantly ↑ survival in αCTLA-4 + αIL-6 vs αCTLA-4 alone	+/±
	αCTLA-4	Balb/c mice<br>CT26 colon carcinoma<br>EAE induction on d3<br>αCTLA-4 d6, 8, 10 and 12	αIL-6 200μg d6, 8, 10 and 12	10	↑ tumour control in αCTLA-4 + αIL-6 vs αCTLA-4 alone	+
	αPD-1	C57BL/6 mice<br>B16.BL6 melanoma<br>EAE induction on d3<br>αPD-1 d3, 6 and 9	αIL-6 200μg d3, 6 and 9	10	Non-significantly ↑ tumour control and significantly ↑ survival in αPD-1 + αIL-6 vs αPD-1 alone	±/+
Liu 2022[102]	αPD-L1	615 mice<br>LA795 lung adenocarcinoma<br>αPD-L1 3 times/w >d12	αIL-6 10mg/kg 3 times/w from d12	5	↑ tumour control in αPD-L1 + αIL-6 vs αPD-L1 alone	+
	αPD-L1	DBA-2J mice	αIL-6 10mg/kg 3 times/w from d9	5	↑ tumour control in αPD-L1 + αIL-6 vs αPD-L1 alone	+

Author	Type ICI	Mice	Immunosuppression	N_{mice}	Results	Effect
		KLN205 lung squamous carcinoma<br>αPD-L1 3 times/w >d9				
Li 2018[107]	αPD-L1	Balb/c mice<br>CT26 colon carcinoma<br>αPD-L1 2 times/w >d7	αIL-6 5mg/kg 2 times/w from d7	10	↑ tumour control in αPD-L1 + αIL-6 vs αPD-L1 alone	+
	αPD-L1	C57BL/6J mice<br>MC38 colon carcinoma<br>αPD-L1 2 times/w >d7	αIL-6 5mg/kg 2 times/w from d7	10	↑ tumour control in αPD-L1 + αIL-6 vs αPD-L1 alone	+
Liu 2017[105]	αPD-L1	Balb/c mice<br>H22 hepatocellular carcinoma + cancer-associated fibroblast injection<br>αPD-L1 weekly >d7,	αIL-6 200µg weekly from d7	10	↑ tumour control and survival in αPD-L1 + αIL-6 vs αPD-L1 alone	+
Mace 2018[108]	αPD-L1	C57BL/6 mice<br>MT5 PDAC<br>αPD-L1 3 times/w from 50-100mm^3	αIL-6 200µg 3 times/w from 50-100 mm^3	5-6	↑ tumour control in αPD-L1 + αIL-6 vs αPD-L1 alone	+
	αPD-L1	C57BL/6 mice<br>Panc02 PDAC<br>αPD-L1 3 times/w from 50-100mm^3	αIL-6 200µg 3 times/w from 50-100mm^3	5-6	Non-significantly ↑ tumour control in αPD-L1 + αIL-6 vs αPD-L1 alone	±
	αPD-L1	C57BL/6 mice<br>KPC-luc PDAC<br>αPD-L1 3 times/w from visible tumours	αIL-6 200µg 3 times/w from visible tumours	5	Similar tumour control in αPD-L1 + αIL-6 vs αPD-L1 alone	±
	αPD-L1	KPC-Brca2 mice with spontaneous PDAC<br>αPD-L1 3 times/w from w5	αIL-6 200µg 3 times/w from w5	5	Proportionally ↓ PanIN3/PDAC lesions in in αPD-L1 + αIL-6 vs αPD-L1 alone after 2 weeks	+

Author	Type ICI	Mice	Immunosuppression	N_{mice}	Results	Effect
Tsukamoto 2018[104]	αPD-L1	C57BL/6 or Balb/c mice<br>MO4-OVA melanoma<br>αPD-L1 d7, 9 and 11	αIL-6 200μg d7, 9 and 11	9	↑ tumour control in αPD-L1 + αIL-6 vs αPD-L1 alone	+
	αPD-L1	C57BL/6 or Balb/c mice<br>B16F10 melanoma<br>αPD-L1 dd5, 7 and 9	αIL-6 200μg d5, 7 and 9	5-10	Non-significantly ↑ tumour control in αPD-L1 + αIL-6 vs αPD-L1 alone	±
	αPD-L1	C57BL/6 or Balb/c mice<br>CT26 colon carcinoma<br>αPD-L1 d4, 7 and 10	αIL-6 200μg d4, 7 and 10	5-10	↑ tumour control in αPD-L1 + αIL-6 vs αPD-L1 alone	+
	αPD-L1	C57BL/6 or Balb/c mice<br>RMA lymphoma<br>αPD-L1 timing unknown	αIL-6 200μg timing unknown	5-10	Similar tumour control in αPD-L1 + αIL-6 vs αPD-L1 alone	±
Li 2022[110]	αPD-1	Balb/c mice<br>CT26 colon carcinoma<br>αPD-1 every 3d from $100m^3$	αIL-6 100μg every 3d from $100m^3$	8	↑ tumour control in αPD-1 + αIL-6 vs αPD-1 alone	+
	αPD-1	C57BL/6 mice<br>MC38 melanoma<br>αPD-1 every 3d from $100m^3$	αIL-6 100μg every 3d from $100m^3$	8	↑ tumour control in αPD-1 + αIL-6 vs αPD-1 alone	+
	αPD-1	C57BL/6 mice<br>B16F10 melanoma<br>αPD-1 every 3d from $100m^3$	αIL-6 100μg every 3d from $100m^3$	8	↑ tumour control in αPD-1 + αIL-6 vs αPD-1 alone	+
	αPD-1	Balb/c mice<br>4T1 breast cancer<br>αPD-1 every 3d from $100m^3$	αIL-6 100μg every 3d from $100m^3$	8	↑ tumour control in αPD-1 + αIL-6 vs αPD-1 alone	+
Ohno 2017[106]	αPD-L1	Balb/c mice<br>CT26 colon carcinoma<br>αPD-L1 d5, 9, 13, 17 and 21	αIL-6R 200μg d5, 9, 13, 17 and 21	6-7	Non-significant ↑ tumour control in αPD-L1 + αIL-6R vs αPD-L1 alone	±

N_{mice}: number of mice per group; αPD-(L)1: programmed cell death (ligand) 1 blockade; αCTLA-4: cytotoxic T lymphocyte-4 blockade; IL: interleukin; αIL-6: interleukin-6 inhibition; cICI: combined immune checkpoint inhibition; EAE: experimental autoimmune encephalomyelitis; PDAC: pancreatic ductal adenocarcinoma

Supplementary table 7: Mouse studies on correlation of Janus (JAK) kinase, mammalian target of rapamycin (mTOR) inhibition or hydroxychloroquine (HCQ) with ICI efficacy

Author	Type ICI	Mice	Immune modulator	N_{mice}	Results	Effect
			**JAK inhibitors**			
Zak 2022[119]	αCTLA-4+ αPD-1	C57BL/6 mice<br>MC38 colon cancer<br>ICI d8 and 15	Ruxolitinib 30 mg/kg, d8-15	10	↑ tumour control in ruxolitinib + cICI vs cICI alone.	+
	αCTLA-4+ αPD-1	BALB/c mice<br>A20 lymphoma<br>ICI every 10d from d8 on.	Ruxolitinib 30 mg/kg, daily from d8 on.	7	↑ tumour control in ruxolitinib + cICI vs cICI alone.	+
	αCTLA-4+ αPD-1	C57BL/6 mice<br>LLC1 lung cancer<br>ICI every 7d from d8 on.	Ruxolitinib 30 mg/kg, daily from d8 on.	5	↑ tumour control in ruxolitinib + cICI vs cICI alone.	+
Benci 2016[120]	αCTLA-4	C57BL/6 mice<br>Res 499 melanoma<br>ICI d5, 8 and 11	Ruxolitinib 60mg/kg d8-12	10	↑ tumour control in ruxolitinib + αCTLA-4 vs αCTLA-4 alone.	+
	αCTLA-4	C57BL/6 mice<br>Res 499 melanoma<br>ICI d5, 8 and 11	Ruxolitinib 60mg/kg d5-9	7	No difference in tumor control between ruxolitinib + αCTLA-4 and αCTLA-4 alone	±
	αCTLA-4	C57BL/6 mice<br>Res 237 breast cancer<br>ICI d5, 8 and 11	Ruxolitinib 60mg/kg d10-14	6	↑ tumour control in ruxolitinib + αCTLA-4 vs αCTLA-4 alone.	+
Lu 2017[121]	αPD-1	C57BL/6 mice<br>PANC02-H7 pancreatic cancer<br>ICI d5, 7, 9, 11, 13 and 15	Ruxolitinib 50mg/kg d5-15	5	↑ tumour control in ruxolitinib + αPD-1 vs αPD-1 alone.	+
			**mTOR inhibitors**			
Langdon 2018[122]	αPD-1 or αPD-L1 or αCTLA-4 monotherapy	C57BL/6 mice<br>MC38 colon carcinoma<br>ICI 2x weekly from d1 on	Vistusertib 15mg/kg Daily from d1 on	15	↑ survival in vistusertib + ICI groups vs ICI alone (for all 3 ICI mono groups).	+

Author	Type ICI	Mice	Immune modulator	N_{mice}	Results	Effect
	αCTLA-4	BALB/c mice<br>CT-26 colon carcinoma<br>ICI 2x weekly from d4 on	Vistusertib 15mg/kg<br>Daily from d4 on	10	Non-significant ↑ tumour control in vistusertib + αCTLA-4 vs αCTLA-4 alone.	±
Bai 2021[123]	αPD-1	C57BL/6 mice<br>SMM103 melanoma<br>ICI d7, 10, 13, 16, 19 and 22	Sirolimus 2mg/kg<br>d7, 10, 13, 16, 19 and 22	4	↑ tumour control in sirolimus + αPD-1 vs αPD-1 alone.	+
Xia 2022[124]	αPD-1	C57BL/6J mice<br>MB49 bladder cancer<br>ICI d0, 3, 6 and 9 from 100 mm^3	Everolimus 2mg/kg, d0-4 and d7-11	4-5	Non-significantly ↓ tumour control with high-dose everolimus + αPD-1 vs αPD-1 alone.	±
	αPD-1	C57BL/6J mice<br>MB49 bladder cancer<br>ICI d0, 3, 6 and 9 from 100 mm^3	Everolimus 0.25mg/kg, d0-4 and d7-11	4-5	Non-significantly ↑ tumour control with low-dose everolimus + αPD-1 vs αPD-1 alone.	±
Song 2022[125]	αPD-1 + radiotherapy	C57BL/6 mice<br>CaSki cervical cancer<br>ICI d11, 13 and 15<br>RT 1x 6Gy 24h after ICI/everolimus.	Everolimus 0.25mg/kg, d11, 13 and 15	8	↑ tumour control with everolimus + αPD-1 vs αPD-1 alone. All groups also received RT.	+
**Hydroxychloroquine**						
Sharma 2020[126]	αPD-1	C57BL6/J mice<br>B16 melanoma<br>ICI d0, 2, 4, 6, 8 and 10 from 50 mm^3	HCQ 60mg/kg d0-11	5	↑ tumour control and survival with HCQ + αPD-1 vs αPD-1 alone.	+
	αPD-1	$BRaf^{CA}$ $Pten^{loxP}$ Tyr::$CreER^{T2}$ mice topical 4-HT induced melanoma<br>ICI d0, 2, 4, 6, 8 and 10 from palpable tumour	HCQ 60mg/kg d0-11	4	↑ tumour control with HCQ + αPD-1 vs αPD-1 alone.	+

Author	Type ICI	Mice	Immune modulator	N_{mice}	Results	Effect
Wabitsch 2021[127]	αPD-1	C57BL/6 mice<br>MC38 colon carcinoma<br>ICI d7, 10 and 15	HCQ 25mg/kg HCQ d5-19	12	↓ tumour control with HCQ + αPD-1 vs αPD-1 alone.	-
	αPD-1	C57BL/6 mice<br>RIL-175 hepatocellular carcinoma<br>ICI d7, 10 and 15	HCQ 25mg/kg HCQ d5-19	8	Non-significantly ↓ tumour control with HCQ + αPD-1 vs αPD-1 alone.	±
	αPD-1	BALB/c mice<br>CT-26 colon carcinoma<br>ICI d7, 10 and 15	HCQ 25mg/kg HCQ d5-19	12	↓ tumour control with HCQ + αPD-1 vs αPD-1 alone.	-
Krueger 2021[128]	αPD-1	C57BL/6J mice<br>B16-PD-L1 melanoma<br>ICI d4, 7, 11 and 14	HCQ 40mg/kg d11-16	14	↓ tumour control with HCQ + αPD-1 vs αPD-1 alone.	-

N_{mice}: number of mice per group; αPD-(L)1: programmed cell death (ligand) 1 blockade; αCTLA-4: cytotoxic T lymphocyte-4 blockade; αIL-6: IL: interleukin-6 inhibition; cICI: combined immune checkpoint inhibition; HCQ: Hydroxychloroquine.

Supplementary table 8: Ongoing clinical trials on immunosuppression for irAE management

ClinicalTrials.gov Identifier	Patients	Treatment arms	ICI efficacy outcomes (all secondary)
NCT04797325	vedolizumab	ICI colitis	none
	prednisolone ± infliximab		
NCT04305145	infliximab	ICI colitis	2yPFS; 2yOS; 2yBORR
	corticosteroids		
NCT04407247	infliximab + corticosteroids	ICI colitis	3mOS
	vedolizumab + corticosteroids		
NCT05335928	abatacept + SoC	ICI myocarditis	none
	placebo + SoC		
NCT05345847	active surveillance	ICI hepatitis	1yPFS
	immediate high-dose corticosteroids		

SoC: standard of care; ICI: immune checkpoint inhibitor; PFS: progression free survival; OS: overall survival; BORR: best overall response rate
